# 

**Published:** 1896-06

**Authors:** 


WOMEN'S EDITION.

WHOLE NUMBER, DXCV. | New Series. JUNE, 1896. | VOL. XXXV. No. 11.

### BUFFALO MEDICAL JOURNAL

**Established 1845 by AUSTIN FLINT, M. D.**

**EDITORIAL STAFF.**

**Women's Edition.**

MAUD JOSEPHINE FRYE, M. D., *Managing Editor.*

ELECTA B. WHIPPLE, M. D.  IDA C. BENDER, M. D.

HELENE KUHLMAN, M. D.

**ASSOCIATE EDITORS:**

JANE W. CARROLL, M. D.  LILLIAN CRAIG RANDALL, M. D.

JANE N. FREAR, M. D.  EVANGELINE CARROLL, M. D.

SCRIPTA FERUNT ANNOS

**EUROPEAN AGENCY**, J. B. BAILLIÈRE & FILS, 19 Rue Hautefeuille, Paris.

*Subscription, if paid in advance, $2.00; otherwise, $2.50. Foreign subscription, $2.50.*

Copyright 1895, by WILLIAM WARREN POTTER, M. D.

Entered at the Post Office at Buffalo, N. Y., as second-class mail matter.

A. T. BROWN PRINTING HOUSE, BUFFALO, N. Y.

**Table of Contents on Page V.**

